# Effectiveness of the conservation areas on the Mornington Peninsula for the common resident shorebird species using citizen science data

**DOI:** 10.1371/journal.pone.0267203

**Published:** 2022-05-04

**Authors:** Udani Abhisheka Wijewardhana, Pragalathan Apputhurai, Madawa Jayawardana, Denny Meyer

**Affiliations:** 1 Department of Health Science and Biostatistics, School of Health Sciences, Swinburne University of Technology, Hawthorn, Victoria, Australia; 2 Peter MacCallum Cancer Centre, Melbourne, Victoria, Australia; 3 Sir Peter MacCallum Department of Oncology, The University of Melbourne, Victoria, Australia; Nanjing Forestry University, CHINA

## Abstract

Conservation areas are critical for biodiversity conservation, but few citizen science studies have evaluated their efficiency. In the absence of thorough survey data, this study assessed which species benefit most from conservation areas using citizen science bird counts extracted from the Atlas of Living Australia. This was accomplished by fitting temporal models using citizen science data taken from ALA for the years 2010–2019 using the INLA approach. The trends for six resident shorebird species were compared to those for the Australian Pied Oystercatcher, with the Black-fronted Dotterel, Red-capped Dotterel, and Red-kneed Dotterel exhibiting significantly steeper increasing trends. For the Black-fronted Dotterel, Masked Lapwing, and Red-kneed Dotterel, steeper rising trends were recorded in conservation areas than in other locations. The Dotterel species’ conservation status is extremely favourable. This study demonstrates that, with some limits, statistical models can be used to track the persistence of resident shorebirds and to investigate the factors affecting these data.

## Introduction

Shorebirds account for around 10% of Australia’s bird species. The majority of Australian shorebirds are declining due to risk factors such as habitat degradation and human activity [1]. Australian shorebirds are found in a wide variety of habitats and wetland types and breed there. Certain species, such as the Sooty Oystercatcher, can be found on both sandy and rocky ocean beaches, as well as the accompanying mudflats. Pied Oystercatchers in Australia prefer tidal mudflats, sandbanks, and sandy ocean beaches. Red-capped Dotterels inhabit tidal or inland saline marshes with broad sandy or muddy coastlines. Masked Lapwings utilise a variety of open habitats, including pasture, sports ovals, and mowed lawns, as well as adjacent wetlands, such as freshwater wetlands and tidal mudflats, typically preferring a mix of grassland and wetland. Both Back-fronted and Red-kneed Dotterels, are found in freshwater marshes [2,3].

Tidal flats in Australia have been damaged by or lost to reclamation, altered water regimes, pollution, sea-level rise and weed invasion [4,5]. Other factors affecting species vulnerability on the Mornington Peninsula are oil spills, dog walkers, introduced rodents, avian predators such as ravens and silver gulls, reptilian predators and weed such as marram grass, sea spurge and sea wheatgrass [6]. Damage of habitat reduces access to food as well as breeding success, with this effect felt more keenly by bird species that nest on the ground as most shorebirds do, with birds spending less time providing care for fledglings when stressed. On the Mornington Peninsula, volunteers from Parks Victoria and BirdLife Australia [7] assist in the monitoring and conservation of nesting shorebirds [8]. Their primary objective is to safeguard Hooded Plovers [9,10], a locally endangered shorebird. Additionally, they manage other threatened species, conserve and enhance habitats, and revegetate. Additionally, reserves are managed to mitigate the impact of wildfire.

Bird Observation and Conservation Australia (now BirdLife Australia) [7] commenced thrice yearly Western Port Bay surveys in late 1973 which provide some support for this hypothesis. Their survey methodology [11,12] focused on the extensive intertidal mudflats of Western Port Bay, with results from these surveys published quite recently [13]. Of the 39 species analysed in the 43-year survey period (1974–2017), 22 species showed declines, including the locally breeding Masked Lapwing. However, it was noted that some species had increased, including the Australian Pied Oystercatcher (many of which breed on French Island, where foxes are absent).

For these reasons, we examined changes in the resident shorebird population on Mornington Peninsula, which is located near Melbourne, Australia. From the mainland to the Bass Strait, this peninsula is bordered on the east by Western Port Bay and on the west by Port Phillip Bay. As explained above, these species exhibit a range of vulnerabilities, with the establishment of conservation zones proving to be an effective strategy of mitigating some of these vulnerabilities. We hypothesised that seven common resident Mornington Peninsula shorebird species which all nest on the ground, Red-capped Plover (*Charadrius ruficapillus*), Red-kneed Dotterel (*Erythrogonys cinctus*), Black-fronted Dotterel (*Elseyornis melanops*), Masked Lapwing (*Vanellus miles*), Australian Pied Oystercatcher (*Haematopus longirostris*) and Sooty Oystercatcher (*Haematopus fuliginosus*), have shown declines in abundance in recent years.

Conservation areas are a key strategy for preserving biodiversity, but few citizen science studies have evaluated the effectiveness of this strategy. Citizen science data from the eBird platform [14] have used to show that creating conservation areas is an effective strategy for retaining species of conservation concern [15]. This conclusion was based on studies of eight tropical forest biodiversity hotspots in Asia, Africa and the Americas, where biodiversity was particularly threatened, and data was particularly scarce. Our study of conservation areas is a much less ambitious project, considering only one section of an important Australian biodiversity hotspot, the Western Port Biosphere Reserve.

A major contributory factor to shorebird decline is habitat loss [16]. Thus, the loss or degradation of a single site could have a profound effect on the persistence of shorebird populations [17]. For species that use many sites throughout the course of their yearly cycle, the site with the worst conditions (e.g., higher mortality or lower carrying capacity) may drive the overall population trend regardless of conservation efforts elsewhere in the species range [18]. Thus, maintaining critical locations during seasonal migration and in non-breeding areas may become a greater conservation priority than protecting breeding grounds, as animals spend a proportionally smaller fraction of their annual cycle moving [19].

The conservation areas of Mornington Peninsula Shire was obtained from the Mornington Peninsula Shire Biodiversity Conservation Plan 2019 [8] that contributes most to biodiversity conservation based on an analysis of remnant native vegetation cover and quality, landscape context and threatened species habitat obtained from Parks Victoria. These areas will benefit the most from actions that are targeted to enhance biodiversity [8]. Birds are better protected if reserves are larger, round or square rather than long and thin, with multiple reserves linked by steppingstones or corridors, and containing at least one large reserve to ensure that local extinctions in small reserves can be replenished [20]. Based on the above it is therefore hypothesised that reductions in the number of shorebirds counts will be greater outside than inside these conservation areas.

Citizen science data plays an increasingly important role in the conservation domain. Continually updated, reliable and comparable biodiversity data is necessary to implement international conservation policy [21]. Citizen science involving non-professionals and professionals as contributors can provide an intensive source of species observation data. As a consequence of the continuous, long running Western Port survey, the significance of Western Port for birds has become widely recognised. As a result, it has become a key monitoring site for Palaearctic and Australasian shorebirds [22]. Conservation actions using citizen science data range from research and monitoring to conservation planning, including tangible conservation actions such as site and habitat management, species management and habitat protection informing law and policy. In traditional citizen science studies, citizens are trained to collect samples using a standardised method to ensure high data quality. The people involved benefit from hands-on learning experiences [23] and improved environmental awareness [24]. The preparation and participation of professional volunteers helps raise community awareness of environmental issues while also contributing to the collection of data that would otherwise be too expensive to acquire [25].

In addition, there has been increasing citizen science effort in recent years which means that trends in absolute numbers of sightings must be treated with caution. The Annual Report 2018–19 for the Port Phillip and Western Port Catchment Management Authority provides an example of the power of citizen science for monitoring the persistence of birdlife in the vicinity of Melbourne, Australia. Between the 2005–06 and 2016 analyses the number of wildlife sightings increased from 437,845 to over 3 million dues to a proliferation of citizen science survey programs. The large amount of data provided by citizen scientists means that more advanced statistical modelling techniques can be applied [26].

The data presented in this study were obtained from Atlas of Living Australia (ALA) which is a validated citizen science database [27]. ALA data management supports the collection and sharing of data with documented quality parameters, with systems developed to implement the process of data collection, digitisation, validation, cleaning and access. The ALA creates filters for their data allowing the removal of duplicate entries, spatially suspect records, and records for which scientific naming of species is not clear [27]. To minimise data biases in citizen science data, we used validated ALA data between 2010 and 2019 for this study. We used citizen science data to examine variations in the reported number of sightings of six resident shorebird species in response to the establishment of conservation areas. Furthermore, we address the rationale for utilising citizen science data in this work and the analysis of these data using advanced statistical modelling processes.

## Methodology

### Statistical models for bird abundance

The development of species distribution models has benefited in the protection of biodiversity by connecting science to policy and decision-making processes. These models have evolved to generate future landscape scenarios based on known and anticipated environmental conditions. However, spatial, or temporal scales can confound inference about changes in species observation data when used to draw conclusions about potential impacts on a different scale. Our data is confined to a relatively small spatial area over a short period of time (2010–2019), and for this reason we only consider temporal models. Annual bird counts are assumed to follow the Poisson distribution [28], and significance is determined when credibility intervals do not contain zero.

The Integrated Laplace Approximation (INLA) method is an approximation tool for fitting Bayesian models for species abundance. INLA is a more resilient alternative to the Markov Chain Monte Carlo (MCMC) based Bayesian analysis technique [29]. The primary advantages of INLA are the ease with which complicated models may be developed and adjusted without requiring complex code and the speed with which inference can be performed, even for temporal issues involving hundreds of thousands of observations [30].

The purpose of this study was to examine the health of resident shorebird species on the Mornington Peninsula using ALA citizen science data and INLA modelling. We examine the effect of conservation reserves on shorebird abundance for seven shorebird species in this study. It is anticipated that the consequences of these variables will vary according to the vulnerability of each shorebird species. Our analysis is separated into two major components. First, we tested whether there are significant differences in the trends for different species, to determine which species are more/less vulnerable. This is done using the entire data set using the species variable with seven categories. Then, we tested the effects of conservation areas for each of the species. This is done for each species using a binary variable to indicate the level of protection for the location of each recorded sighting.

### Data sources and study area

Records of annual data for resident shorebirds for the Mornington Peninsula from 2010 to 2019 were obtained from the Atlas of Living Australia database. The Atlas of Living Australia database gathers basic data on bird abundance and distribution at a variety of temporal scales. Much of this geocoded observational data were gathered during systematic surveys made by trained volunteers or qualified biologists [27,31].

To obtain only the validated data, we have filtered the data as follows. Excluded spatially suspect records, records based on scientific name quality, records with additional spatial quality issues, duplicate records, records based on location uncertainty, records with unresolved user annotations, records that are environmental outliers, records based on record type and records pre-1700. We have included present-only records from eBird Australia [14], BirdLife Australia (Birdata) and Victorian Biodiversity Atlas (VBA). Since we have considered each month to aggregate the data in annual level, we have excluded data which has no year available.

The resident shorebird species included in this study are: Black-fronted Dotterel, Masked Lapwing, Red-capped Dotterel, Red-kneed Dotterel, Australian Pied Oystercatcher and Sooty Oystercatcher. In this study, considered the total number of bird sightings recorded in each year for each of the above seven resident shorebird species, separately for conservation and other areas.

The Mornington Peninsula is a unique place when it comes to biodiversity. It is home to a wide range of plants and animals, including species of regional, state, national and international significance. The Mornington Peninsula is built of complex geological formations, resulting in diverse landforms and habitat types. The major habitat types on the Peninsula include central hills, waterways, wetlands, north central plains, sandy beaches and dunes, cliffs and headlands, rocky shores, mudflats, saltmarsh, mangrove swamps and estuaries. Approximately 10% of the peninsula land is protected within parks or reserves, including a national park, a state park, state conservation reserves, local bushland reserves and foreshore coastal reserves. These remnant areas of bushland provide an important refuge for the diverse range of plants and animals on peninsula.

The Mornington Peninsula forms part of the Western Port Biosphere Reserve (WPBR), which covers five Local Government Areas around Western Port Bay and carries out projects to test ways of balancing conservation with development. The Western Port wetlands are included in the Ramsar List of Wetlands of International Importance and are the primary reason why the United Nations Education, Scientific and Cultural Organisation (UNESCO) declared the Western Port catchment as one of only four active biosphere reserves in Australia, and one of only 701 in the world [8]. The Western Port Ramsar Site was designated as a Wetland of International Importance under the Convention on Wetlands of International Importance (Ramsar Convention) in 1982 [32], which regularly supports a high diversity and large number of waterbirds [33].

The Mornington Peninsula National Park is the largest reserve on the peninsula with inland and coastal components. Mornington Peninsula National Park is the most visited National Park in Victoria, with intensively used recreation nodes at Portsea, Sorrento, and Cape Schanck [8]. Because it is located close to residential areas and is such a popular holiday destination, the Mornington Peninsula is subject to a wide range of threats and pressures for shorebird species. The shorebirds in this area are especially threatened by human disturbance, recreational activities, and predation. However, the Mornington Peninsula Shire undertakes a range of conservation programs to protect and enhance biodiversity and reduce the impact of threats posed by environmental weeds, pest animals and habitat loss.

### Statistical analysis

We fitted temporal models for the number of annual sightings recorded for the above resident shorebirds on the Mornington Peninsula between 2010–2019. A Bayesian hierarchical modelling approach was used to conveniently account for parameter uncertainty and potential temporal dependence. We have considered temporal models with Poisson and Negative Binomial distributions [28]. In most cases better fit, measured using the lowest Deviance Information Criterion (DIC) and Watanabe-Akaike information criterion (WAIC) was obtained for the Poisson distribution as defined below;

$$P(Y=k)=\frac{\lambda^{k}e^{-\lambda }}{k!},$$

with *λ*>0 defined as the mean of this distribution and *k* the number of sightings in a single year. As described below we define a general hierarchical model for annual total shorebird counts (*Y*) in terms of categorical predictor variables with the annual trend.

For a categorical variable, such as species or level of protection, the general hierarchical model can be described as follows, where *Y*_*tj*_ is the annual shorebird counts for a specific category (*j*) in a particular year *(t*) and *f*(*y*_*tj*_|*λ*_*tj*_) is the Poisson distribution defined above with relevant mean parameters (*λ*_*tj*_).


$$Y_{tj}∼f(y_{tj}|\lambda_{tj})\;where\;t=1,2,\ldots ,10\;and\;j=1:\#of\;categories$$

We include the categorical variables in the model by way of dummy variables (υ), with one of the categories chosen as the reference category. The interaction between year and these dummy variables tells us about the difference in slope between these categories and the reference category and allows a test of significant.

For species *j* the general link function for the expected annual total shorebird counts is given below with *δ* ‘s included as species parameters. The Australian Pied Oystercatcher was chosen as the reference category in this analysis because this species showed the least change in the number of annual sightings over time (See Fig 2).


$$\log\left(\lambda_{tj}\right)=\beta_{0}+\beta_{1}t+\delta_{0j}\upsilon_{j}+\delta_{1j}(\upsilon_{j}*t)+\varphi_{t}+u_{t}\;where\;t=1,2,\ldots ,10$$

andj=1:#ofspeciesexcludingthereferencecategory

Depending on whether or not a sighting occurred in a conservation area the general link function for the expected annual total shorebird counts is given below with *δ* ‘s included as protection parameters (See Fig 3).


$$\log\left(\lambda_{tp}\right)=\beta_{0}+\beta_{1}t+\delta_{0p}\upsilon_{p}+\delta_{1p}(\upsilon_{p}*t)+\varphi_{t}+u_{t}\;where\;t=1,2,\ldots ,10$$

$$and\;\upsilon_{p}=1\;for\;a\;protected\;area,\;0\;for\;an\;unprotected\;area$$

As before *φ*_*t*_ allows for a temporal random effect with first-order autoregressive dependence and the random error term is represented by *u*_*t*_. All these models were implemented using the R-INLA [34] approach in R 4.1.3 [35]. We have implemented a shiny app for annual temporal models using R-INLA which can be accessed from https://github.com/uwijewardhana/UDMTA.

## Results

### Descriptive Statistics

**Fig 1:**
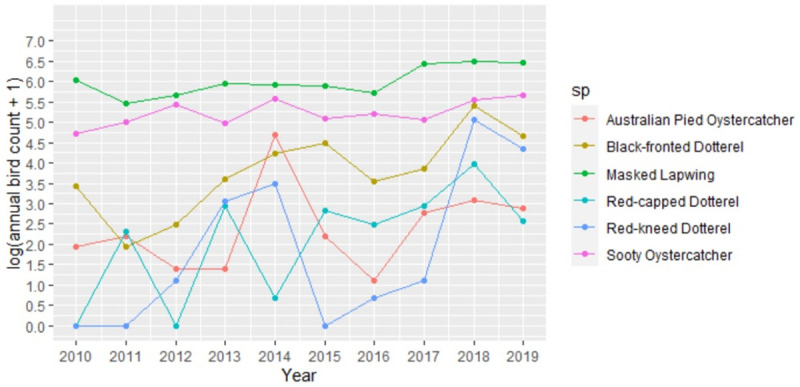
Annual species counts for the Mornington Peninsula from 2010–2019.

Table 1 shows that the Atlas of Living Australia data was mainly sourced from eBird together with the estimated annual growth rates in the number of sightings and the percentage of sightings recorded in conservation areas for each species. Fig 1 illustrates the changes in annual sightings recorded for the seven resident shorebirds over the period 2010–2019 on a logarithmic scale.

**Table 1:** Total number of birds in citizen science sightings extracted from Atlas of Living Australian by Data Source (2010–2019).

Species	BirdLife	eBird	Victorian Biodiversity Atlas	Total counts	Estimated growth per annum (%) 2010–2019	% Sightings in conservation areas
**Australian Pied Oystercatcher**	3	84	103	190	4.7	10%
**Black-fronted Dotterel**	110	519	15	644	26.6	54%
**Masked Lapwing**	1715	2328	213	4256	9.2	58%
**Red-capped Dotterel**	8	129		137	14.6	18%
**Red-kneed Dotterel**	8	279		287	44.5	88%
**Sooty Oystercatcher**	293	1632	1	1926	6.4	87%

### Species effects

**Fig 2:**
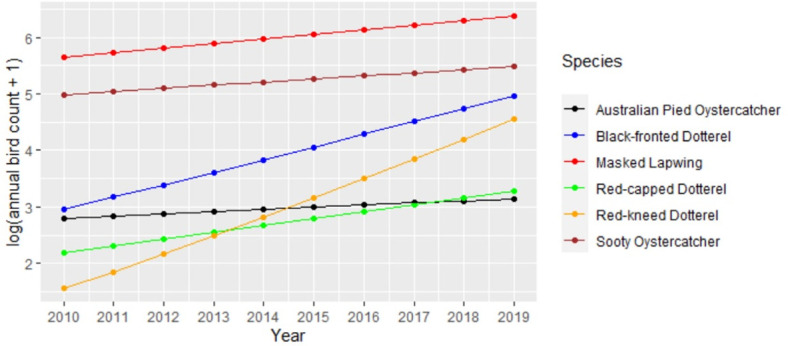
Model predictions of log transformed trend models for annual species sightings for the Mornington Peninsula.

Table 2 provides the results for a model testing whether there was significant difference in the trends for the various species. In this model the Australian Pied Oystercatcher has been chosen as the reference level because it showed the least change in the number of annual sightings and showed the weakest trend over time. When compared to the Australian Pied Oystercatcher there is a significantly stronger trend for the Black-fronted Dotterel, Red-capped Dotterel and Red-kneed Dotterel with estimated growth rates of 19%, 9% and 32% per annum. This significance is indicated by the credibility intervals for their *δ*_1*j*_ estimates which do not contain zero. Fig 2 illustrates the above trends on a logarithmic scale.

**Table 2:** Fixed effects for species trends using the Australian Pied Oystercatcher as the reference species (significant effects bolded).

Coefficients	Mean	SD	95% Credibility Interval	
			0.025quant	0.975quant
*Intercept*				
**Australian Pied Oystercatcher—reference (*β*_0_)**	2.701	0.293	2.147	3.32
**Black-fronted Dotterel (*δ*_*01*_*)***	-0.03	0.203	-0.424	0.371
**Masked Lapwing (*δ*_*02*_*)***	**2.854**	**0.167**	**2.535**	**3.19**
**Red-capped Dotterel (*δ*_*03*_*)***	**-0.759**	**0.328**	**-1.415**	**-0.127**
**Red-kneed Dotterel (*δ*_*04*_*)***	**-1.734**	**0.323**	**-2.38**	**-1.111**
**Sooty Oystercatcher (*δ*_*05*_*)***	**2.223**	**0.171**	**1.896**	**2.566**
***Trend by Year***				
**Australian Pied Oystercatcher—reference (*β*_1_)**	0.04	0.044	-0.053	0.124
**Black-fronted Dotterel (*δ*_*11*_*)***	**0.188**	**0.029**	**0.131**	**0.245**
**Masked Lapwing (*δ*_*12*_*)***	0.042	0.025	-0.008	0.091
**Red-capped Dotterel (*δ*_*13*_*)***	**0.091**	**0.045**	**0.004**	**0.179**
**Red-kneed Dotterel (*δ*_*14*_*)***	**0.317**	**0.041**	**0.238**	**0.398**
**Sooty Oystercatcher (*δ*_*15*_*)***	0.015	0.026	-0.036	0.066

### Effects of conservation areas

**Fig 3:**
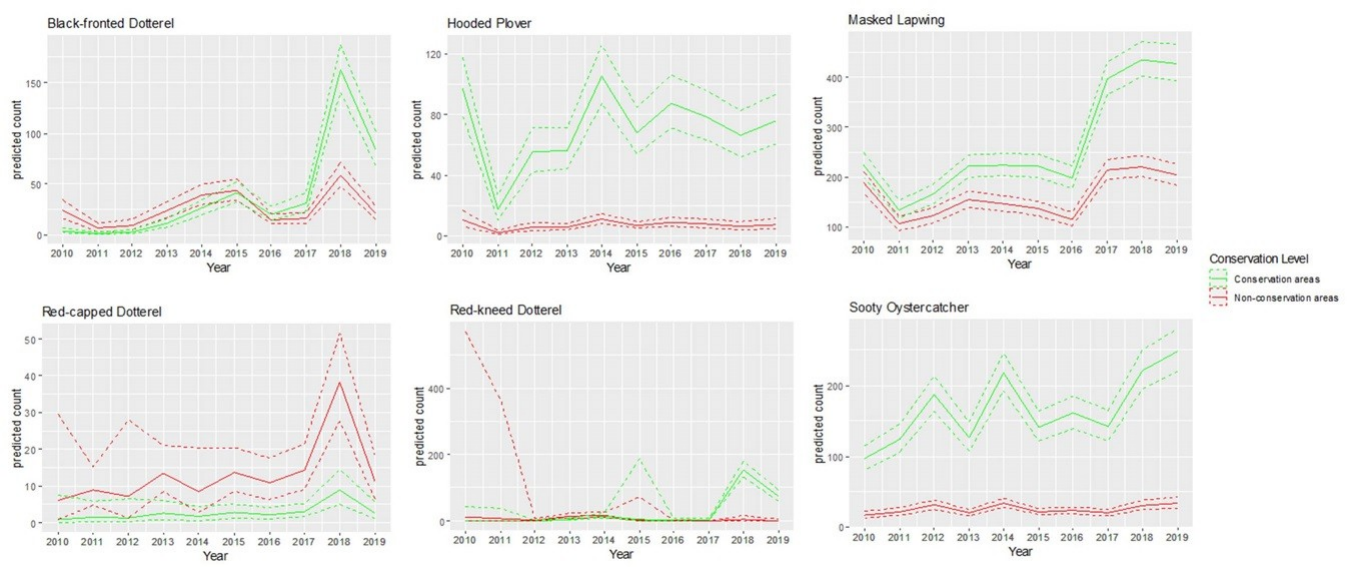
Model predictions of trends for the local shorebird species significant in conservation areas.

There are many ongoing conservation programs on the Mornington Peninsula. Our results in Table 3 show that the conservation areas are particularly important for the resident shorebirds including locally threatened Sooty Oystercatcher. However, although the bird counts for the Black-fronted, Red-capped and Red-kneed Dotterels were initially low, the results show that the latest trend is starting to reverse this effect with significantly greater growth seen in conservation areas. Fig 3 illustrates these upward trends while also emphasising the obvious growth in Masked Lapwing numbers.

**Table 3:** Fixed effects of conservation level models using the areas do not include in conservation sites as the reference level with significant effects bolded.

Species	Coefficients	Mean	SD	0.025quant	0.975quant
**Australian Pied Oystercatcher**	Intercept	1.589	1.034	-0.529	3.676
	year	0.107	0.16	-0.22	0.433
	conservation area	-3.519	2.9	-9.771	1.612
	year: conservation area	0.192	0.325	-0.392	0.885
**Black-fronted Dotterel**	Intercept	2.613	0.808	1.151	4.452
	year	0.078	0.121	-0.199	0.296
	**conservation area**	**-2.166**	**0.293**	**-2.753**	**-1.603**
	**year: conservation area**	**0.354**	**0.038**	**0.28**	**0.431**
**Masked Lapwing**	Intercept	4.841	0.286	4.35	5.517
	year	0.04	0.042	-0.056	0.117
	conservation area	0.111	0.073	-0.032	0.254
	**year: conservation area**	**0.063**	**0.011**	**0.042**	**0.084**
**Red-capped Dotterel**	Intercept	1.786	0.677	0.397	3.102
	year	0.121	0.092	-0.068	0.301
	**conservation area**	**-1.759**	**0.919**	**-3.655**	**-0.047**
	year: conservation area	0.036	0.117	-0.187	0.272
**Red-kneed Dotterel**	Intercept	2.918	3.517	-4.796	9.547
	year	-0.405	0.484	-1.375	0.602
	**conservation area**	**-5.325**	**1.192**	**-7.965**	**-3.294**
	**year: conservation area**	**1.015**	**0.234**	**0.624**	**1.539**
**Sooty Oystercatcher**	Intercept	2.991	0.25	2.452	3.448
	year	0.041	0.039	-0.031	0.125
	**conservation area**	**1.695**	**0.153**	**1.4**	**1.999**
	year: conservation area	0.03	0.023	-0.017	0.076

## Discussion

The purpose of this study was to examine the effect of species and conservation areas on the numbers of the Mornington Peninsula’s common locally nesting shorebird species. This was accomplished through the use of data from the Atlas of Living Australia and Poisson temporal models fitted using the INLA approach. We were interested in determining whether the rate at which reported numbers change over time varies by species and protection areas. Our findings pose several critical conservation challenges.

While the Australian Pied Oystercatcher population has expanded dramatically in Western Port over the last 40 years [25], our data indicates that the neighbouring Mornington Peninsula had just a 4.7% yearly increase from 2010 to 2019. Rather than that, our results indicate that when compared to the Australian Pied Oystercatcher, the Black-fronted Dotterel, Red-capped Dotterel, and Red-kneed Dotterel have all shown a considerable rising tendency. The growth in observer numbers explains why citizen science data collection effort has increased throughout the years. Possible explanations for the increase in Dotterel counts include an increased interest in these birds. For example, in 2010, “The Friends of the Hooded Plover Mornington Peninsula” began collecting data on the Red-capped Dotterel. This may have resulted in a recent surge in survey activity for these species.

Declines in population were reported for both the Masked Lapwing and the Red-capped Dotterel up to 2009 [36]. However, the 9.2% annual growth rate for ALA sightings for the Masked Lapwing observed in this study and the 14.6% annual growth rate for the Red-capped Dotterel suggest that these trends have since reversed, or did not apply to the different habitats sampled in our study.

Coastal environments are the most vulnerable for resident shorebirds on the Peninsula, particularly for shorebirds with limited distributions such as the Sooty Oystercatcher. We discovered a strong favourable effect for the locally threatened Sooty Oystercatcher in our conservation area models. The increased numbers of Sooty Oystercatchers may be explained by their locally threatened status, which necessitates regular surveys and the establishment of conservation areas.

In addition, this study has found that the percentage increase for the numbers of Black-fronted Dotterel, Red-kneed Dotterel and Red-capped Dotterel has been stronger in conservation areas than other areas on Mornington Peninsula since 2010, and the same has been true for the Masked Lapwing. Contributing factor are likely to include the control of introduced predators such as Red Fox (*Vulpes vulpes)*, Cat (*Felis catus)*, Black Rat (*Rattus rattus*,) and Domestic Dog (C*anis familiaris*) in conservation areas. The core habitat for Red-kneed and Black-fronted Dotterels is inland wetlands. The study period (2010–2019) coincided with severe inland drought, with the inland becoming increasingly dry through the study period. This may have been the cause of the increase in the Mornington Peninsula area as inland wetlands dried up. The increasing trends may also mean that the monitoring of the local Dotterels is more pervasive than for other birds in conservation areas, producing the higher counts observed in these areas. However, for Pied Oystercatcher, there is no evidence to suggest that conservation areas are successful for increasing the number of shorebird counts.

Among other habitats, the Australian Pied Oystercatcher, Masked Lapwing, and Red-capped Plover make extensive use of tidal mudflats. Previously, only peripheral data existed on species that frequent freshwater habitats (Black-fronted and Red-kneed Dotterels), as well as rocky or sandy coastlines (Sooty Oystercatcher). The majority of conservation areas on the Mornington Peninsula that are important to shorebirds are located on picturesque beaches, tidal mudflats, and reserves with freshwater habitats and sandy coasts. This habitat disparity between the Mornington Peninsula and previously published research could account for the majority of the changes mentioned above. The ALA sighting data used in this study do not corroborate previously reported positive trends for the Australian Pied Oystercatcher by [25]. Additionally, Masked Lapwing numbers were originally reported to be falling, but our study indicates that this is no longer the case. However, the prior study concentrated on a different habitat type (intertidal mudflats in the Western Port survey), rather than the diverse range of habitat types seen on the Mornington Peninsula. Additionally, other changes have occurred since 2009, including the establishment of new conservation areas and fox eradication campaigns, which may have influenced the trends documented in this study.

Citizen science data is recommended for large geographic area studies, such as Australian shorebird analysis, and seasonal data, such as pest eradication analysis, but is less reliable for small geographic area studies, such as Mornington Peninsula analysis. Through citizen science databases, observer data is made publicly available with the collaboration of the observers. The majority of sensitive data and structured survey data are not accessible to the general public. As a result, our study utilised validated citizen science ALA data. However, there are legitimate worries about the detection bias inherent in citizen science data [37], which is a limitation of this study.

Along with the nearby Edithvale-Seaford Wetlands, the Eastern treatment plant is part of the Carrum Wetlands Important Bird Area (IBA) and supports many bird species of regional, state, national and international conservation significance. This has not included as a conservation area and may be affect for the species performance. We have not included this Eastern treatment plan which is also a limitation of this study.

## Conclusions

Temporal models fitted with INLA provide versatile and useful frameworks for statistically modelling count data. While citizen science data has limitations, particularly when comparing relative citizen science bird counts of various species, there are evident benefits to using citizen science data to monitor endangered species citizen science counts. Citizen science data are often of higher quality for these species, at least in terms of survey data obtained in conservation areas, and it would be difficult to obtain sufficient data from other sources. However, increasing counts of other birds in conservation areas, such as Dotterels, indicates an increase in citizen science effort for less endangered species, which bodes well for future conservation efforts.

According to the findings of this study, local shorebirds benefit from the presence of conservation areas. However, it appears improbable that conservation areas would be established at many of the sites with high levels of human activity, suggesting that better conservation outcomes may result from achieving desirable shorebird abundance targets. It has been suggested by the findings of this study that, in the future, statistical models may be useful for addressing challenges associated with monitoring the persistence of resident shorebirds and for exploring factors that have an impact on these data.

## Supporting information

S1 Dataset Original dataset. (CSV)

S2 Dataset Species effect model dataset. (CSV)

S3 Dataset Conservation area model dataset. (CSV)

S1 File R code. (R)
